# Association of Rheumatoid Arthritis and Hepatitis B Infection: A Nationwide Nested Case-Control Study From 1999 to 2009 in Taiwan

**DOI:** 10.1097/MD.0000000000003551

**Published:** 2016-05-06

**Authors:** Ching-Sheng Hsu, Hui-Chu Lang, Kuang-Yung Huang, Hans Hsienhong Lin, Chien-Lin Chen

**Affiliations:** From the Division of Gastroenterology, (C-SH, HHL), Department of Internal Medicine, Taipei Tzu Chi Hospital, Buddhist Tzu Chi Medical Foundation, New Taipei; School of Post-Baccalaureate Chinese Medicine (C-SH), Tzu Chi University, Hualien, Taiwan; School of Medicine (C-SH, K-YH, HHL, C-LC), Tzu Chi University, Hualien; Institute of Hospital and Health Care Administration (H-CL), National Yang-Ming University, Taipei; Division of Allergy (K-YH), Immunology, and Rheumatology, Department of Internal Medicine, Dalin Tzu Chi Hospital, Buddhist Tzu Chi Medical Foundation; Department of Life Science and Institute of Molecular Biology (K-YH), National Chung Cheung University, Chiayi; and Division of Gastroenterology (C-LC), Department of Internal Medicine, Hualien Tzu Chi Hospital, Buddhist Tzu Chi Medical Foundation, Hualien, Taiwan.

## Abstract

Rheumatoid arthritis (RA) is a disorder with altered immunologic function and increased risks of infection, while the association between HBV and RA remains largely unknown.

To determine the prevalence and risk of HBV infection in patients with RA, 2 cohort datasets were sourced from Taiwan's National Health Insurance Research Database to capture National Health Insurance claims data between 1999 and 2009. One set was a specially requested RA subject's dataset extracted from the whole 23 million beneficiaries, and a total of 38,969 aged ≧18 years RA subjects were identified (RA cohort). The other one was a randomly selected 1 million patients’ longitudinal dataset, and from which an additional 701,476 aged ≧18 years non-RA subjects were identified (non-RA cohort). An epidemiological approach was used to compare the prevalence and risk for HBV infection between RA and non-RA subjects.

During the followed interval between 1999 and 2009, 3260 in RA cohort and 63,588 in non-RA cohort had a diagnosis of HBV infection. The annual age- and sex-standardized prevalence of HBV infection in the RA cohort was generally higher than that in the non-RA cohort. The RA patients had a higher HBV period prevalence than did the non-RA subjects (RA vs. non-RA = 69.9 vs. 60.1 cases per 1000 subjects). Compared with the non-RA cohort, the RA cohort had an increased risk of HBV infection after adjustment for potential prognostic factors (1.13, 95% CIs: 1.08–1.17).

RA patients are characterized by an increased risk of HBV infection than non-RA subjects.

## Abstract 2

Supplemental Digital Content is available in the text

## INTRODUCTION

Hepatitis B virus (HBV) infection is the leading cause of chronic liver disease worldwide.^1^ HBV may interact with hepatocytes and the immune system, leads to liver fibrosis progression, cirrhosis, decompensation, and hepatocellular carcinoma (HCC)^2,3^ in patients with chronic HBV infection. Moreover, chronic HBV infection has been linked to several rheumatic manifestations,^4,5^ and implied to play a pathogenic role in rheumatoid arthritis (RA).^6,7^

RA is a systemic inflammatory disease involving altered immunologic function, and RA patients have increased risks of several types of bacterial and viral infection.^8,9^ Although RA patients receiving biologic agents for immunomodulatory treatment have been associated with the reactivation of HBV infection^10^ that may result in liver failure and death,^11^ the association of HBV infection and RA remains largely unknown. A few studies have investigated the prevalence of HBV infection in RA patients; most of the these studies have been limited to small sample sizes and specific populations,^7,12–16^ focused on patients who received biologic agents,^11^ and could not attain conclusive results.^17^ Whether RA has a pathogenic association with HBV infection remains unanswered. Considering the global prevalence of HBV infection and the increasing use of biologic agents in the treatment of RA patients, a comprehensive understanding of the association between HBV and RA has become necessary in managing both conditions.

In this study, we compared the prevalence and incidence of HBV infection between RA and non-RA subjects. Specifically, we hypothesized that RA may affect the risk of HBV infection. Therefore, we analyzed Taiwan's National Health Insurance Research Database, which included all 23 million beneficiaries in Taiwan, and used an epidemiologic approach to compare the prevalence and risk of HBV infection between RA and non-RA subjects.

## METHODS

### Ethics Statement

This study was conducted in accordance with the Helsinki Declaration. Deidentified data released by Taiwan's National Health Research Institutes (NHRI), which are available to the public for research, were used. Information that could be used to identify patients or care providers, including medical institutions and physicians, was scrambled before being sent to the NHRI for database construction and is further scrambled before being released to each researcher. Thus, the remaining health information cannot be used to identify a person. All researchers who wish to use Taiwan's National Health Insurance Research Database (NHIRD) and its data subsets are required to sign a written agreement declaring that they have no intention to obtaining information that could potentially violate the privacy of patients or care providers.

### Study Design and Data Sources

We conducted a nationwide case-control study by obtaining RA cases (RA cohort) and controls (non-RA cohort) from NHI claims data recorded in the NHIRD between 1999 and 2009. The NHIRD has been described in detail in previous studies.^18,19^ In brief, the NHIRD contains the registration data of all people who have been beneficiaries of the NHI program, which covered 99% of Taiwan's population and approximately 22,600,000 people in 2007. The NHRI maintains the NHIRD and provides it to scientists in Taiwan for research purposes. The accuracy of diagnoses of major diseases in the NHIRD, such as stroke and acute coronary syndrome, has been validated previously.^20^

### Study Population

We identified a total of 38,969 RA subjects (RA cohort) from a specially requested RA subject dataset that was extracted from all beneficiaries between January 1, 1999 and December 31, 2009. All RA subjects were required to have been diagnosed with RA (International Classification of Diseases, Ninth Revision, Clinical Modification [ICD-9-CM] code 714.0x) ≥2 times and to be ≥18 years of age on the date of first diagnosis. The accuracy of RA diagnoses was confirmed by both specific ICD-9-CM codes and inclusion in the Registry for Catastrophic Illness Patient Database (RCIPD), a subset of the NHIRD that contains records of all adult RA patients who were diagnosed with RA ≥2 times and met the 1987 American College of Rheumatology diagnostic criteria each time.^21^ We excluded patients who were diagnosed with RA only once during the study interval, <18 years of age when first diagnosed with RA, first diagnosed with RA after July 1, 2009, or not registered in the RCIPD.

The non-RA cohort comprised 701,476 subjects who were ≥18 years of age, had no RA diagnosis, and were identified from a dataset of 1 million randomly selected insurance beneficiaries included in the Registry for Beneficiaries of the Taiwan NHI program between 1999 and 2009. This dataset was provided by the NHRI, which asserted that there are no statistically significant differences in sex distribution between the randomly sampled beneficiaries and all beneficiaries in the NHI program.

### Main Outcome Measurements

Patients with HBV infection were defined as those diagnosed with HBV infection (ICD-9-CM codes 070.2, 0.70.3, and V02.61) ≥2 times, those who received 1 HBV infection diagnosis and 1 measurement of HBeAg (14034C, 14035C, and 27035B) or anti-HBe (14036C and 27036B), or hospitalized patients who were admitted with a primary diagnosis of HBV infection. The standardized prevalence, incidence rate of HBV infection, and incidence rate ratio (IRR) were compared between the RA and non-RA subjects. The risk of HBV infection in the RA subjects versus that in the non-RA subjects was calculated after adjustment for potential prognostic factors.

### Covariate Assessment

To determine the effect of RA on the risk of HBV infection, it is crucial to consider the influences of known prognostic factors. We thus extracted variables frequently associated with HBV infection. These baseline prognostic factors included the patients’ age in years, sex, biologic agents for RA, nucleoside analogues for HBV infection, and comorbidities present in the 180-day period prior to the initial RA diagnosis date defined by ICD-9-CM codes (diabetes mellitus [DM], obesity, HIV infection, ischemic heart disease [IHD], alcohol-related illness, chronic obstructive pulmonary disease [COPD], and liver cirrhosis). Because the smoking status was unavailable for analyses, COPD was selected as a proxy for cigarette smoking. All ICD-9-CM codes used in the study are provided in Supplementary Table 1.

Information regarding patients’ medications, including the use of biologic agents for RA and nucleoside analogues for HBV infection, was obtained from the pharmacy prescription database. The reliability of the retrieved information was verified independently by 2 statisticians. Biologic agents for RA that were available in Taiwan during the study interval included etanercept, adalimumab, rituximab, abatacept, actemra, and golimumab; and nucleoside analogues for HBV infection included lamivudine, adefovir, entecavir, telbivudine, and tenofovir.

### Statistical Analysis

SAS 9.3 for Windows (SAS Institute Inc, Cary, NC) was used to perform the statistical analyses in this study. The demographic data, clinical characteristics, and comorbidities were compared between the RA and non-RA cohorts. The data were presented as percentages for categorical variables as well as means with standard deviations for continuous variables unless mentioned otherwise, and were analyzed using the Pearson *χ*^2^ test, Fisher exact test, Student *t* test, and Wilcoxon rank-sum test, where appropriate.

The prevalence of HBV infection was determined by dividing the number of HBV infections by the total number of RA or non-RA subjects. The incidence was determined by dividing the number of newly detected HBV infections by the total number of RA or non-RA subjects. The incidence density rate was determined by dividing the number of newly detected HBV infections by the total observation period of RA or non-RA subjects calculated in patient-years. The observation period of RA and non-RA cohorts was tracked from the date of selection until the end of the study or until loss to follow-up (i.e., withdrawal from the health insurance program) to identify HBV infection events. For patients who experienced multiple HBV infection events, only the first event was included. The IRR for RA compared with non-RA was calculated by dividing the average number of newly detected HBV infections per total patient-years in the RA cohort by the average number of newly detected HBV infections per total patient-years in the non-RA cohort.

The standardized prevalence or incidence was defined as the ratio of the observed to the expected prevalence or incidence, respectively, in the cohorts. The expected prevalence or incidence of HBV infection was calculated by summing all numbers (prevalence) or all person-time (incidence rate) in the cohort, dividing the sum into strata by age and sex, and then multiplying the stratum-specific number or person-time by the corresponding stratum-specific prevalence or incidence rate of the entire Taiwan population in 2000.

To compare the risk of HBV infection between the RA and non-RA cohorts, we used logistic regression models with different combinations of covariates, including the initial diagnosis year of HBV infection, sex, age, the use of biologic agents, the use of antiviral agents, and preexisting comorbidities (DM, obesity, alcohol-related illness, HIV infection, IHD, COPD, and liver cirrhosis). Because differences in health behaviors between patients with and without RA could influence the association between RA and outcome events, we used influenza vaccination as a proxy of health behaviors for the sensitivity analysis.

## RESULTS

### Demographic Data of the RA and Non-RA Cohorts

**FIGURE 1:**
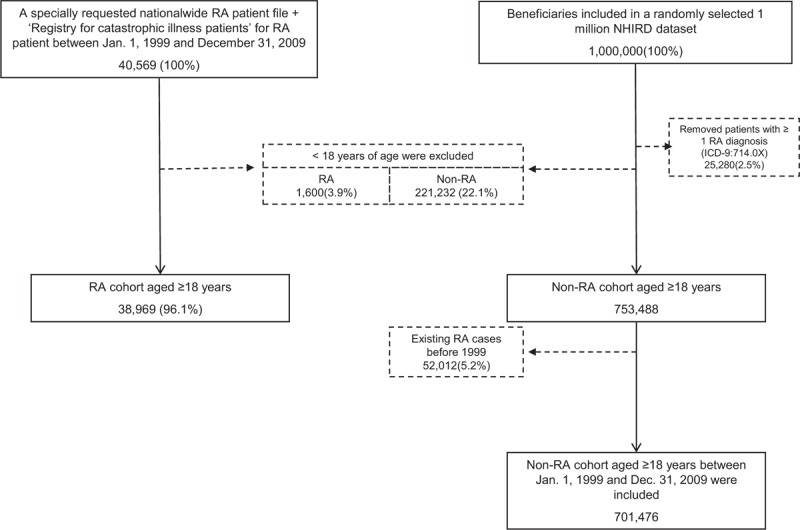
Selection of study patients.

A total of 38,969 potentially eligible RA patients aged ≧18 years with a registered record in the RCIPD and 701,476 non-RA subjects aged ≧18 years during the same study period were enrolled (Figure 1). Among them, 3620 RA patients and 63,588 non-RA subjects with a diagnosis of HBV infection were identified (Figure 1). The demographic characteristics, antiviral agents, biologic agents, and comorbidities of the study population are presented in Table 1. Compared with the non-RA subjects, the RA subjects were predominantly female and older, used biologic and antiviral agents more frequently, and had a higher likelihood of comorbidities including DM, IHD, cerebrovascular diseases, COPD, and liver cirrhosis (Table 1).

**TABLE 1:**
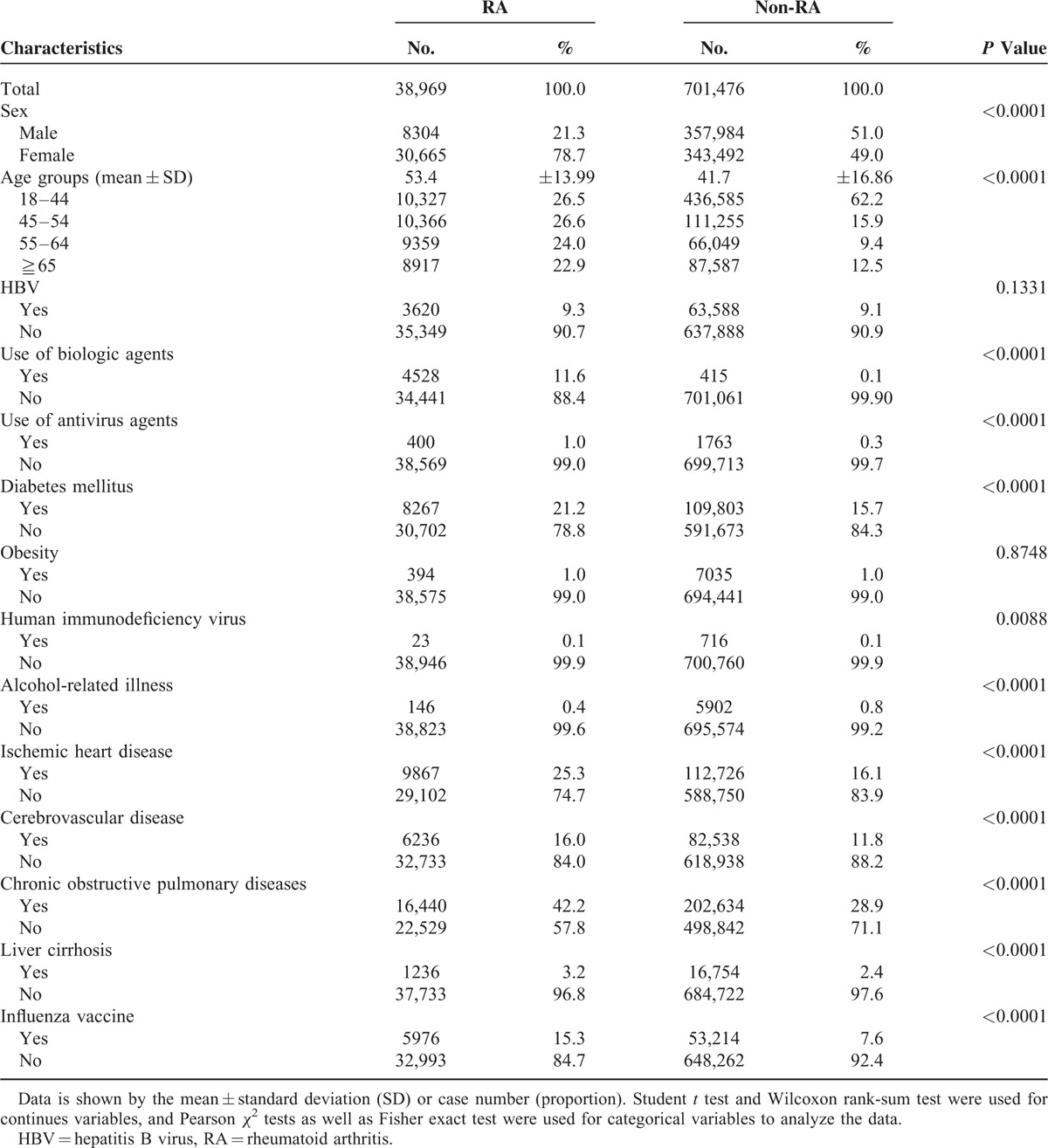
Characteristics of RA and Non-RA Subjects in the Study Population

### Standardized Prevalence and Incidence of HBV Infection

The annual age- and sex-standardized prevalence of HBV infection in the RA subjects was generally higher than that in the non-RA subjects between 1999 and 2009. The RA patients had a higher HBV period prevalence than did the non-RA subjects (period standardized prevalence of HBV infection of RA vs. non-RA = 69.9 vs. 60.1 cases per 1000 subjects). Moreover, among the men, the prevalence of HBV infection in the RA subjects was higher than that in the non-RA subjects, whereas the prevalence was similar between the 2 groups among the women (Supplementary Figure 1; period standardized prevalence of HBV infection of RA vs. non-RA in men: 81.9 vs. 54.3 cases per 1000; in women: 67.3 vs. 65.9 cases per 1000).

The annual age- and sex-standardized incidence of newly detected HBV infection in RA subjects was higher than that in non-RA subjects during the period between 1999 and 2009 (period standardized incidence for HBV infection of RA vs. non-RA = 17.1 vs. 7.34 cases per 1000). Moreover, the standardized incidence of HBV infection in RA subjects was higher than that in non-RA subjects among both the men and women (Supplementary Figure 2; period standardized incidence of HBV infection of RA vs. non-RA in men: 22.3 vs. 7.0 cases per 1000; in women: 16.4 vs. 7.7 cases per 1000). The HBV IRR for RA versus non-RA subjects was 1.33 (1.25–1.42) among all subjects, 1.74 (1.53–1.97) among the men, and 1.17 (1.09–1.26) among the women (Table 2, Supplementary Tables 2–4).

**TABLE 2:**
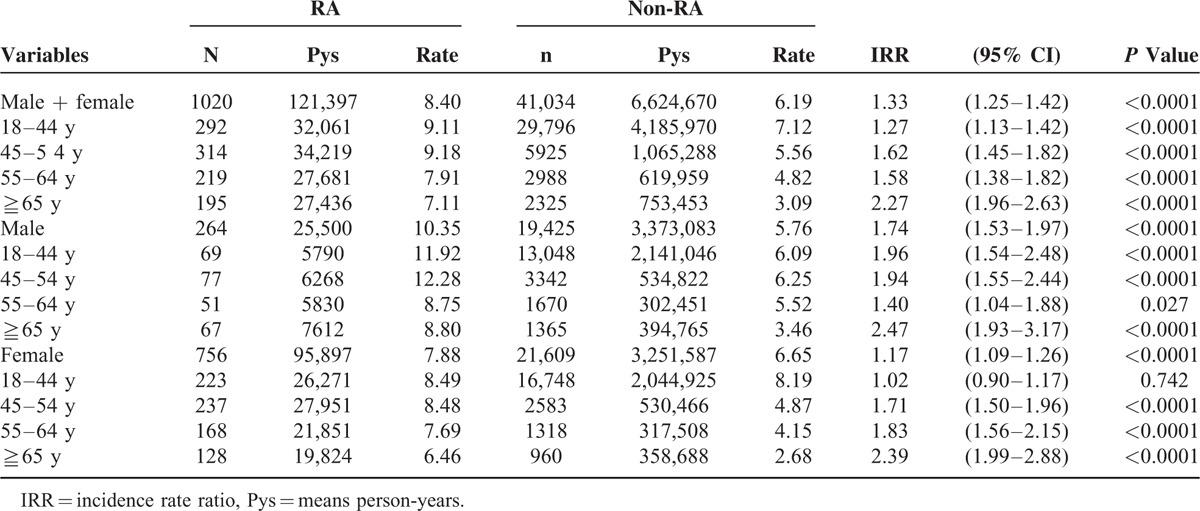
Incidence Rate Ratio for HBV Infection Among the RA and Non-RA Cohorts Followed From 2000 to 2009 in Taiwan

### Multivariable Analysis

To examine the association of HBV infection with RA, we performed multivariate analyses with different logistic regression models after adjustment for covariate factors (Table 3). Compared with the non-RA cohort, the RA cohort had an increased risk of HBV infection after adjustment for sex, age, DM, obesity, alcohol-related illness, HIV infection, IHD, cerebrovascular diseases, COPD, and liver cirrhosis (adjusted odds ratio, 1.13; 95% confidence interval, 1.08–1.17; *P* <0.0001). Because differences in health behaviors between patients with and without RA could influence the association of RA with its outcome events, we used influenza vaccination as a proxy of health behaviors, and examined the association of HBV infection and RA after adjustment for influenza vaccination; the conclusion remained unchanged.

**TABLE 3:**
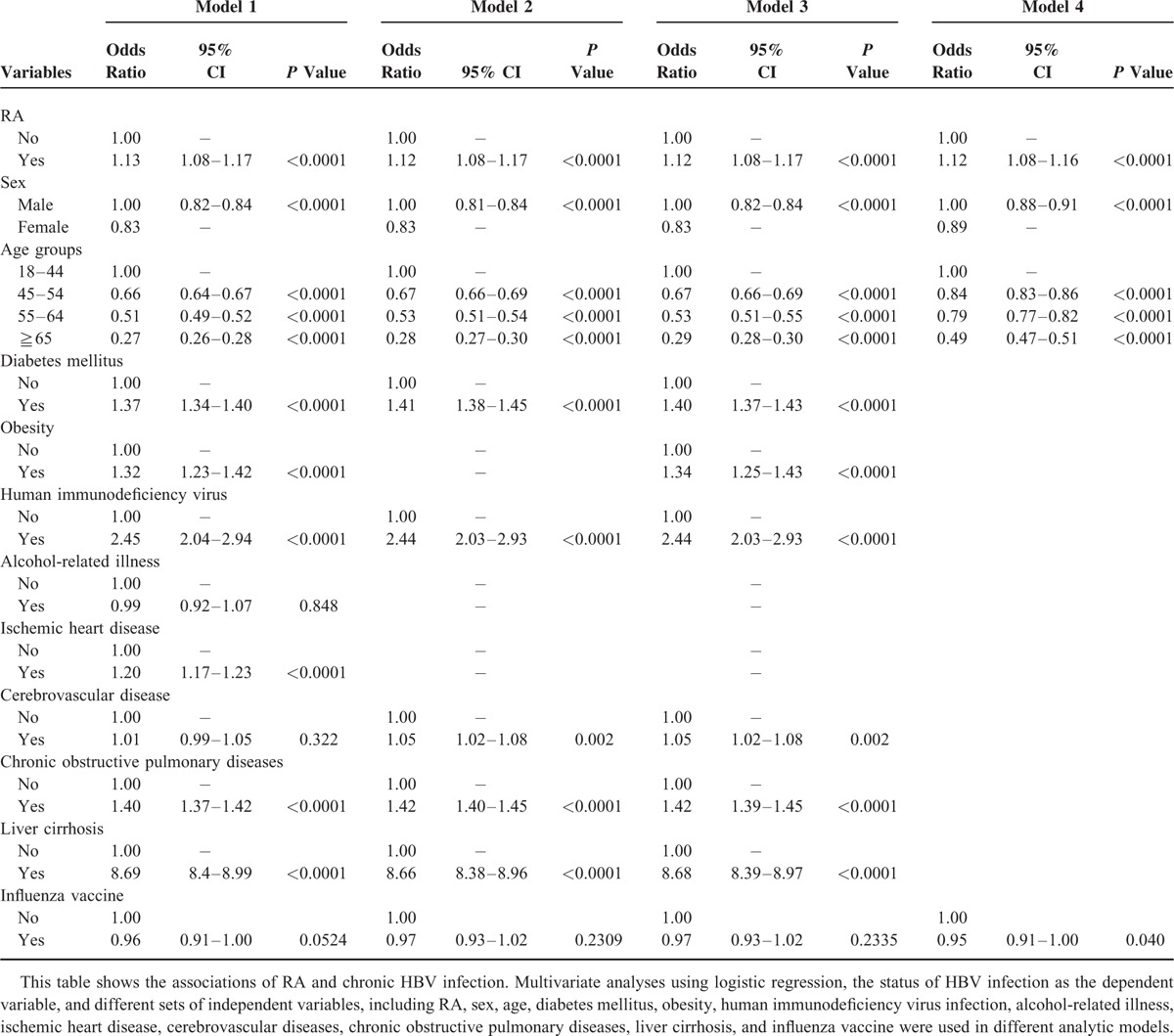
Multivariate Analyses Examining the Association of RA and HBV Infection

## DISCUSSION

Taking advantage of the accurate RA registry in Taiwan, we determined the national prevalence and incidence of HBV infection in RA patents, and found that RA subjects had a higher HBV prevalence (69.9 cases per 1000) and incidence (17.1 cases per 1000) than did matched non-RA subjects. A salient finding from this national-scale data was that RA was associated with a 13% increased risk of HBV infection in the general population after adjustment for known prognostic factors.

HBV infection has been linked to systemic autoimmune diseases,^4^ and patients with chronic HBV infection may have various extrahepatic manifestations such as serum-sickness-like syndrome, vasculitis, skin rash, arthritis, and glomerular manifestations.^22^ However, most studies that examined the association between HBV infection and RA have been limited to a few HBV cases and specific populations;^7,12–16,23^ whether RA has a pathogenic association with HBV infection remains undetermined.^17^ This study involved using a nationwide database from Taiwan, an HBV-endemic country, to examine the association of HBV with RA. The major strengths of this study are the large sample size, sufficient number of HBV cases, and capability to clarify the association between HBV and RA. In addition to demonstrating a higher prevalence, incidence, and risk of HBV infection among RA patients, this study provided strong population-based evidence supporting the hypothesis that HBV may play a pathogenic role in RA.^6,7^

Although the molecular mechanisms underlying the pathogenesis of HBV-associated extrahepatic manifestations remain largely unknown, HBV antigenemia-related immune-complex-mediated injury, including the deposition of immune complexes containing HBV viral antigens (HBsAg or HBeAg) and their antibodies (anti-HBs and anti-HBe) in the synovial tissues of HBV-associated arthritis,^6,24,25^ has been proposed as the cause.^22,26^ Moreover, the polymorphic residues of major histocompatibility complex (MHC) class II molecules in RA patients may bind HBsAg amino acid peptide sequences,^27^ and arthritis associated with chronic HBV infection may resolve after successful antiviral treatment,^28,29^ further supporting a direct link between RA and HBV infection. However, future studies are required to clarify these molecular mechanisms.

This study has the limitations of retrospective studies in attributing causality. First, because our observations excluded subjects <18 years of age when first diagnosed with RA, who were first diagnosed with RA after July 1, 2009, or who were not registered in the RCIPD, certain selection biases may exist, and caution must be taken in generalizing our results to some characteristic populations. Coding errors, misclassifications and measurement errors in the diagnosis of HBV infection might have occurred and potentially under- or overestimate the HBV infection rate, and the use of steroid or biologic agents would overestimate the infection rate. Moreover, as Taiwan's National Health Insurance does not reimburse a regular HBV examination in subjects who do not have a risk or contact history for HBV infection, the HBV infection rate from Taiwan's National Health Insurance Research Database will be lower than previous reports from Taiwan. Second, data on baseline serum immunological, biochemical, virologic profiles, pathological characteristics and the severity of rheumatoid arthritis that are likely associated with the link between RA and HBV infection were generally lacking from insurance claims and were not considered when examining the association between RA and HBV infection risk. Third, information on the lifestyle, health concerns, and medication compliance of the patients was unavailable. However, we thus used influenza vaccination as a surrogate for health behavior, and found that the effects of RA and HBV infection risk are independent of influenza vaccination. Fourth, although age and the period of observation may affect the values of IRR, we examined the IRR between RA and non-RA group among different age strata, and found that the values of IRR remained statistically significant among different age-year starta. Of note, we compared the IRR between different periods of observation (Supplementary Table 2 & 3), and found that the values of IRR during the period 2000 to 2004 were more pronounced than those between 2005 and 2009. Because subjects included between 2000 and 2004 have a longer HBV infectious period than those between 2005 and 2009, a longer infectious period may be a reason for the more pronounced IRR observed. Last, the use of the data from all 23 million beneficiaries in Taiwan, and the fact that the data were originally collected for a different purpose increase the validity of the findings and indicate that these results are likely applicable to the whole population and different subgroups of RA patients.

## CONCLUSIONS

Our study indicates that RA subjects have a higher risk of HBV infection regardless of other confounders. RA patients had a higher period prevalence, incidence, and incidence rate ratio for HBV infection than did the non-RA subjects. Further research on the pathogenesis of the association between RA and HBV infection and its clinical influence on the disease outcomes is necessary.

## Supplementary Material

Supplemental Digital Content
