# Supplementary material for: Association of Rheumatoid Arthritis and Hepatitis B Infection: A Nationwide Nested Case-Control Study From 1999 to 2009 in Taiwan
Source: Medicine (Baltimore). 2016 May 6;95(18):e3551. doi: 10.1097/MD.0000000000003551 (PMC4863786; doi:10.1097/MD.0000000000003551)
Supplement: Supplemental Digital Content [file medi-95-e3551-s001.doc]

**
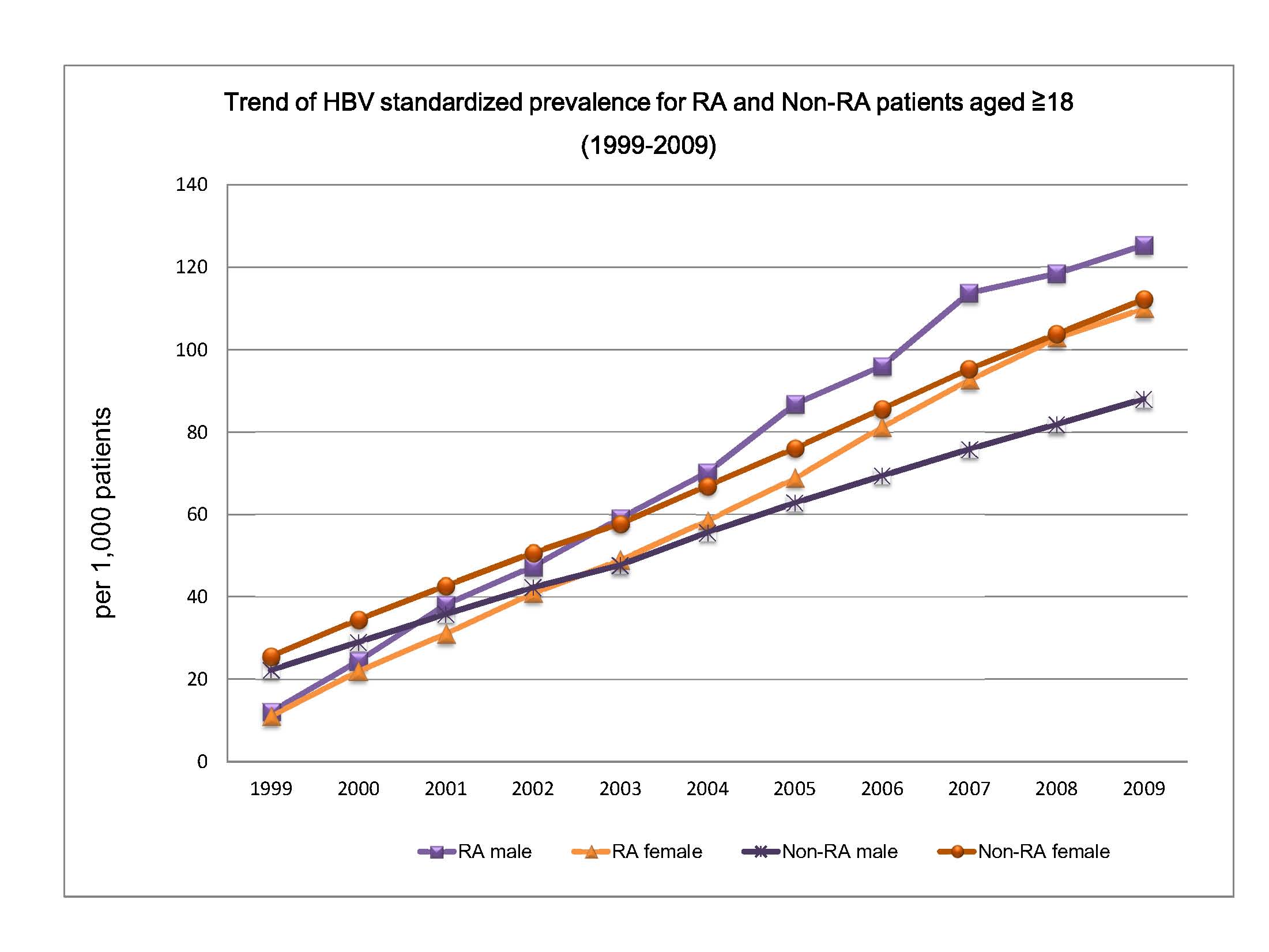
**

**
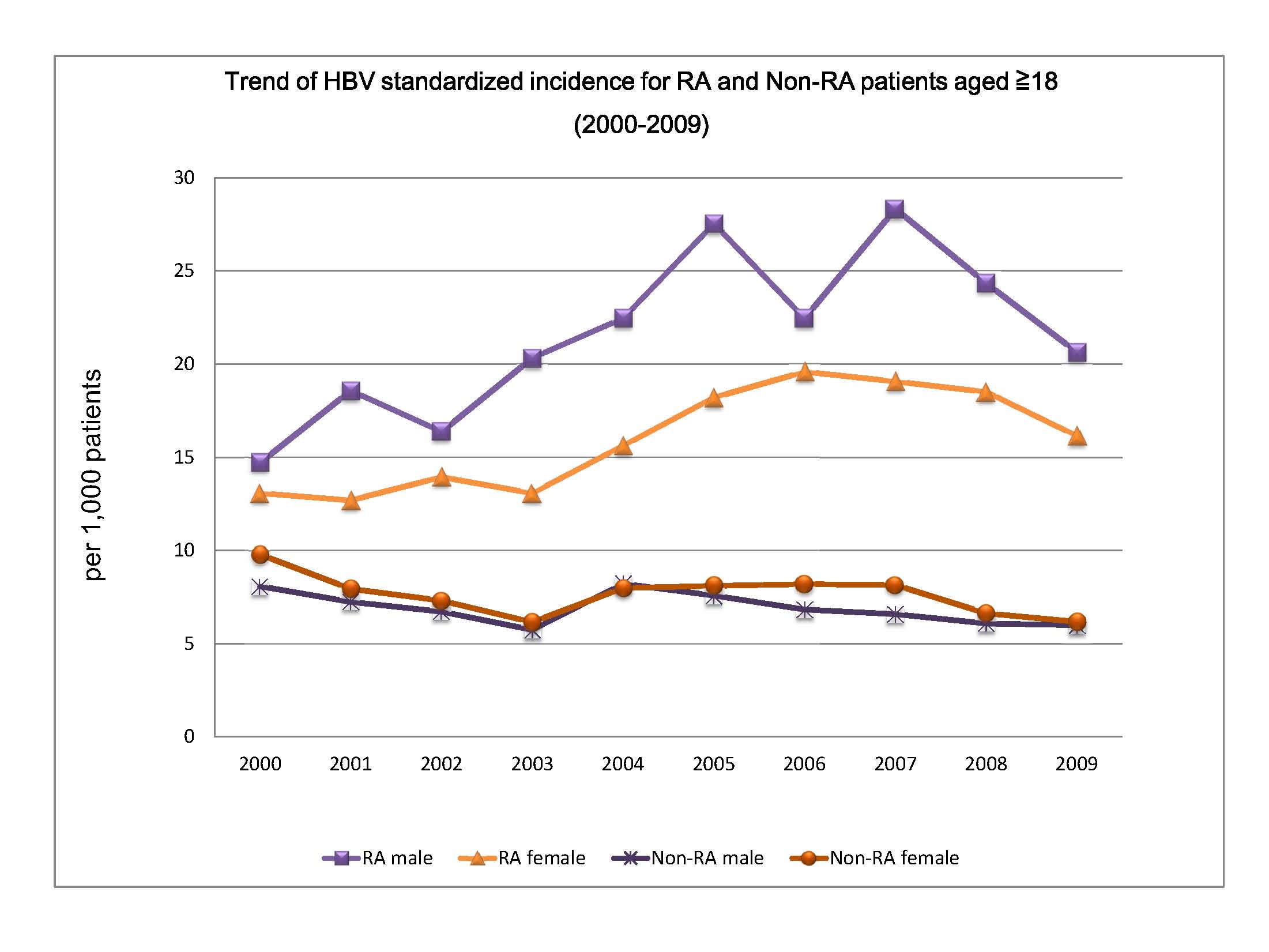
**

**Supplementary Table 1. The International Classification of Diseases, Ninth Revision, Clinical Modification (ICD-9-CM) codes used in the study.**

| **Comorbidities** | **ICD-9-CM codes** |
| --- | --- |
| Diabetes mellitus | 250 |
| Obesity | 278, 278·0, 278·00, 278·01 |
| Human immunodeficiency virus infection | 042, V08, 079·53 |
| Ischemic heart diseases | 410-414 |
| Alcohol related illness | 291, 303, 305.0, 571.0, 571.1, 571.2, 571.3 |
| Chronic obstructive pulmonary diseases | 490-496 |
| Liver cirrhosis | 571·2, 571·5, 571·6 |

**Supplementary Table2. Incidence rate ratio for HBV infection among the RA and non-RA cohorts followed from 2000 to 2004 in Taiwan**

| Variables | RA | | | |  | Non - RA | | | |  |  |  | P-Value |
| --- | --- | --- | --- | --- | --- | --- | --- | --- | --- | --- | --- | --- | --- |
|  | n | N | Pys | Rate |  | n | N | Pys | Rate |  | IRR | (95%CI) |  |
| Male + Female | 704 | 13260 | 32578 | 21.61 |  | 41034 | 701477 | 3452092 | 11.89 |  | 1.81 | (1.68-1.95) | <.0001 |
| 18-44 y | 203 | 3411 | 8565 | 23.70 |  | 29796 | 436585 | 2166543 | 13.75 |  | 1.72 | (1.5-1.98) | <.0001 |
| 45-54 y | 218 | 3640 | 8877 | 24.56 |  | 5925 | 111256 | 551450 | 10.74 |  | 2.28 | (1.99-2.61) | <.0001 |
| 55-64 y | 155 | 2978 | 7445 | 20.82 |  | 2988 | 66049 | 324045 | 9.22 |  | 2.21 | (1.88-2.61) | <.0001 |
| ≧65 y | 128 | 3231 | 7690 | 16.64 |  | 2325 | 87587 | 410053 | 5.67 |  | 2.94 | (2.46-3.51) | <.0001 |
| Male | 175 | 2850 | 6833 | 25.61 |  | 19425 | 357984 | 1758823 | 11.04 |  | 2.28 | (1.96-2.65) | <.0001 |
| 18-44 y | 45 | 626 | 1505 | 29.90 |  | 13048 | 222257 | 1104295 | 11.82 |  | 2.53 | (1.89-3.39) | <.0001 |
| 45-54 y | 52 | 661 | 1598 | 32.54 |  | 3342 | 56337 | 278306 | 12.01 |  | 2.71 | (2.06-3.56) | <.0001 |
| 55-64 y | 37 | 638 | 1560 | 23.72 |  | 1670 | 32693 | 159393 | 10.48 |  | 2.08 | (1.48-2.92) | <.0001 |
| ≧65 y | 41 | 925 | 2170 | 18.89 |  | 1365 | 46697 | 216830 | 6.30 |  | 3.00 | (2.2-4.1) | <.0001 |
| Female | 529 | 10410 | 25744 | 20.55 |  | 21609 | 343493 | 1693268 | 12.76 |  | 1.61 | (1.47-1.75) | <.0001 |
| 18-44 y | 158 | 2785 | 7060 | 22.38 |  | 16748 | 214328 | 1062249 | 15.77 |  | 1.42 | (1.21-1.66) | <.0001 |
| 45-54 y | 166 | 2979 | 7279 | 22.81 |  | 2583 | 54919 | 273144 | 9.46 |  | 2.40 | (2.05-2.81) | <.0001 |
| 55-64 y | 118 | 2340 | 5886 | 20.05 |  | 1318 | 33356 | 164652 | 8.00 |  | 2.50 | (2.07-3.02) | <.0001 |
| ≧65 y | 87 | 2306 | 5520 | 15.76 |  | 960 | 40890 | 193224 | 4.97 |  | 3.17 | (2.55-3.95) | <.0001 |

**Supplementary Table 3. Incidence rate ratio for HBV infection among the RA and non-RA cohorts followed from 2005 to 2009 in Taiwan**

| Variables | RA | | | |  | Non - RA | | | |  |  |  | P-Value |
| --- | --- | --- | --- | --- | --- | --- | --- | --- | --- | --- | --- | --- | --- |
|  | n | N | Pys | Rate |  | n | N | Pys | Rate |  | IRR | (95%CI) |  |
| Male + Female | 316 | 12640 | 30522 | 10.35 |  | 19093 | 679536 | 3300122 | 5.79 |  | 1.70 | (1.52-1.91) | <.0001 |
| 18-44 y | 89 | 3016 | 7659 | 11.62 |  | 11601 | 347198 | 1700618 | 6.82 |  | 1.65 | (1.33-2.04) | <.0001 |
| 45-54 y | 96 | 3468 | 8645 | 11.10 |  | 3714 | 138098 | 676075 | 5.49 |  | 1.94 | (1.58-2.38) | <.0001 |
| 55-64 y | 64 | 2980 | 7055 | 9.07 |  | 1987 | 76214 | 370281 | 5.37 |  | 1.56 | (1.2-2.02) | <.0001 |
| ≧65 y | 67 | 3176 | 7163 | 9.35 |  | 1791 | 118026 | 553148 | 3.24 |  | 2.76 | (2.15-3.54) | 0.0008 |
| Male | 89 | 2923 | 6795 | 13.10 |  | 9069 | 347628 | 1685831 | 5.38 |  | 2.27 | (1.83-2.82) | <.0001 |
| 18-44 y | 24 | 567 | 1419 | 16.91 |  | 4913 | 178571 | 877034 | 5.60 |  | 3.02 | (2.02-4.51) | <.0001 |
| 45-54 y | 25 | 730 | 1758 | 14.22 |  | 2051 | 69358 | 338277 | 6.06 |  | 2.25 | (1.51-3.37) | <.0001 |
| 55-64 y | 14 | 690 | 1558 | 8.99 |  | 1092 | 38246 | 184810 | 5.91 |  | 1.19 | (0.66-2.16) | <.0001 |
| ≧65 y | 26 | 936 | 2061 | 12.62 |  | 1013 | 61453 | 285710 | 3.55 |  | 3.28 | (2.19-4.92) | 0.5571 |
| Female | 227 | 9717 | 23727 | 9.57 |  | 10024 | 331908 | 1614291 | 6.21 |  | 1.48 | (1.29-1.69) | <.0001 |
| 18-44 y | 68 | 2449 | 6240 | 10.90 |  | 6688 | 168627 | 823584 | 8.12 |  | 1.22 | (0.95-1.57) | 0.1136 |
| 45-54 y | 71 | 2738 | 6887 | 10.31 |  | 1663 | 68740 | 337797 | 4.92 |  | 2.01 | (1.57-2.56) | <.0001 |
| 55-64 y | 50 | 2290 | 5497 | 9.10 |  | 895 | 37968 | 185471 | 4.83 |  | 1.81 | (1.35-2.42) | <.0001 |
| ≧65 y | 41 | 2240 | 5103 | 8.03 |  | 778 | 56573 | 267438 | 2.91 |  | 2.69 | (1.96-3.7) | <.0001 |

*Pys means Person-years

**Supplementary Table 4. Incidence rate ratio for HBV infection among the RA and non-RA cohorts followed from 2000 to 2009 in Taiwan**

| Variables | RA | | | |  | Non - RA | | | |  |  |  | P-Value |
| --- | --- | --- | --- | --- | --- | --- | --- | --- | --- | --- | --- | --- | --- |
|  | n | N | Pys | Rate |  | n | N | Pys | Rate |  | IRR | (95%CI) |  |
| Male + Female | 587 | 25931 | 122,428 | 4.79 |  | 20391 | 701476 | 6,732,149 | 3.03 |  | 1.57 | (1.45-1.71) | <.0001 |
| 18-44 y | 149 | 6428 | 32,409 | 4.60 |  | 13582 | 436585 | 4,271,753 | 3.18 |  | 1.44 | (1.22-1.69) | <.0001 |
| 45-54 y | 176 | 7128 | 34,609 | 5.09 |  | 3448 | 111255 | 1,077,517 | 3.20 |  | 1.59 | (1.37-1.85) | <.0001 |
| 55-64 y | 129 | 5950 | 27,828 | 4.64 |  | 1804 | 66049 | 625,765 | 2.88 |  | 1.58 | (1.32-1.90) | <.0001 |
| ≧65 y | 133 | 6425 | 27,582 | 4.82 |  | 1557 | 87587 | 757,114 | 2.06 |  | 2.33 | (1.95-2.78) | <.0001 |
| Male | 158 | 5786 | 25,730 | 6.14 |  | 12155 | 357984 | 3,409,558 | 3.56 |  | 1.71 | (1.46-2.00) | <.0001 |
| 18-44 y | 33 | 1194 | 5,897 | 5.60 |  | 8053 | 222258 | 2,166,128 | 3.72 |  | 1.51 | (1.07-2.12) | 0.019 |
| 45-54 y | 45 | 1398 | 6,320 | 7.12 |  | 2070 | 56336 | 541,274 | 3.82 |  | 1.86 | (1.39-2.50) | <.0001 |
| 55-64 y | 28 | 1324 | 5,848 | 4.79 |  | 1072 | 32693 | 305,432 | 3.51 |  | 1.32 | (0.90-1.93) | 0.1593 |
| ≧65 y | 52 | 1870 | 7,665 | 6.78 |  | 960 | 46697 | 396,723 | 2.42 |  | 2.81 | (2.12-3.71) | <.0001 |
| Female | 429 | 20145 | 96,699 | 4.44 |  | 8236 | 343492 | 3,322,591 | 2.48 |  | 1.78 | (1.61-1.96) | <.0001 |
| 18-44 y | 116 | 5234 | 26,512 | 4.38 |  | 5529 | 214327 | 2,105,625 | 2.63 |  | 1.65 | (1.37-1.99) | <.0001 |
| 45-54 y | 131 | 5730 | 28,290 | 4.63 |  | 1378 | 54919 | 536,242 | 2.57 |  | 1.80 | (1.51-2.16) | <.0001 |
| 55-64 y | 101 | 4626 | 21,981 | 4.59 |  | 732 | 33356 | 320,333 | 2.29 |  | 1.99 | (1.62-2.45) | <.0001 |
| ≧65 y | 81 | 4555 | 19,917 | 4.07 |  | 597 | 40890 | 360,391 | 1.66 |  | 2.42 | (1.92-3.06) | <.0001 |

*Pys means Person-years

**NOTE.** HBV was defined strictly as those who received one HBV infection diagnosis and one measurement of HBeAg (14034C, 14035C, and 27035B) or anti-HBe (14036C and 27036B).
